# Comparison of analgesic and anxiolytic effects of nitrous oxide in burn wound treatment: A single-blind prospective randomized controlled trial

**DOI:** 10.1097/MD.0000000000018188

**Published:** 2019-12-20

**Authors:** Lin Li, Qiong Pan, Le Xu, Renqin Lin, Jiaxi Dai, Xinyan Chen, Meiyun Jiang, Zhaohong Chen

**Affiliations:** a Department of Burns; b Department of Nursing, Fujian Medical University Union Hospital; c Fujian Medical University Union Clinical Medical Institute, Fuzhou, Fujian Province, China.

**Keywords:** burn patients, nitrous oxide, pain, titration

## Abstract

**Aim:**

We compared the effects of 50% N_2_O and N_2_O titration in burn management to alleviate pain and anxiety associated with burn dressing.

**Methods:**

In this single-blind prospective randomized controlled trial, 70 stable adult burn patients were randomized to 2 groups during May 2015 to January 2016. The experimental group was titrated with N_2_O ranging from 30% to the ideal sedation concentration before dressing change until the end. The control group was treated with 50% N_2_O 2 minutes before dressing change until the end. Pain, anxiety, vital signs, and the highest concentrations of N_2_O inhaled were recorded at 1 minute before N_2_O inhalation (T0), dismantling of outer (T1), inner dressings (T2), debridement (T3), drug-smearing (T4), bandaging (T5), and 10 minutes after completion of the procedure (T6).

**Results:**

The pain and anxiety scores in the experimental group performed significantly less than the control group during T2-T6. The systolic blood pressure in T2 and the heart rate at T2 and T3 varied significantly between the 2 groups. The highest N_2_O concentrations of the experimental group were mainly 60% to 70% at T2 (87.9%), T3 (87.9%), and T4 (81.8%).

**Conclusion:**

N_2_O titration significantly reduced pain and anxiety in burn patients, with minimal side effects.

## 1 Introduction

Pain is the most common clinical symptom in burn patients, which is especially more intense and unbearable in wound dressing.^[1]^ Pain not only affects the prognosis and outcome of patients, but triggers psychological and social distress, including depression, anorexia, insomnia, and post-traumatic stress disorder.^[2–4]^ In addition, repeated pain procedures often create anticipatory anxiety in burn patients.^[5]^ Anxiety induced by a painful experience with a poor dressing change can lead to bad compliance with rehabilitation, increased pain, and a loss of trust in the burn team.^[6]^ If left untreated, anxiety can also escalate into fear, insomnia, depression, and helplessness, which may cause patients to be psychologically unable to cope with their disease.^[7]^ Therefore, exploration of safe and effective analgesic and anxiolytic drugs is the goal of burn care.

Opioids are still the main treatment for moderate and severe burn pain. However, opioids can cause a multitude of side effects with a high incidence, such as constipation, itching, nausea, respiratory depression, and so on.^[8]^ The incidence of side effects of opioids can be as high as 76% to 92% in patients with acute pain.^[9]^ Nonsteroidal anti-inflammatory drugs (NSAIDs)are widely used analgesics. They are weak when used alone, but have synergistic effects with opioids.^[10]^ Side effects, particularly gastrointestinal bleeding, may limit its use in severely burned patients.^[11]^

The use of opioids, NSAIDs to control pain and anxiety in China is not extensive due to lack of clinical awareness.^[2]^ Patients often are not treated effectively because physicians in China are worried about the possible complications.^[12]^ Nitrous oxide (N_2_O), a colorless, slightly fragrant inhalation anesthetic may be more appropriate than the aforementioned drugs for acute burn pain in China due to its safety, and rapid analgesic and anxiolytic action.

The blood gas distribution coefficient of N_2_O is 0.47, which is extremely stable in the blood without combination with any substances. N_2_O quickly diffuses through the alveolar-capillary membrane and acts within 30 to 40 seconds after inhalation.^[13]^ The analgesic effect peaks in 5 minutes. Transient breathing cessation eliminates 99% of the N_2_O from the alveoli, and patients fully recover after administration of 100% pure oxygen for about 5 minutes.^[13]^ Nitrous oxide is not metabolized via liver, and 99% is expelled via lungs, and 0.004% through gastrointestinal tract.^[13]^ Two major types of established N_2_O analgesia are available. The first type involves 50% N_2_O (Entonox) inhalation, which is used to induce painless labor or abortion,^[14,15]^ endoscopy,^[16]^ and in the emergency department.^[17]^ Rapid inhalation alleviates pain and anxiety promptly. However, this approach may increase discomfort in adult patients, and the probability of adverse reactions such as nausea and vomiting.^[13]^ The second type of analgesia entails N_2_O titration, which slowly increases the dosage from 0% to 70% to determine the appropriate dose. It provides individualized treatment of patients using the minimum effective dose, without excessive sedation. The treatment is mainly used in dental procedures.^[18,19]^

Evidence suggests that N_2_O ameliorated burn pain and anxiety in pediatric population^[20–22]^ in contrast to conflicting findings in the adult population. Yuxiang L found that 50% N_2_O has a positive effect on dressing pain relief.^[12]^ However, do Vale et al^[23]^ reported that the use of 65% N_2_O resulted in no additional benefit. Moreover, a British survey of nurses in 11 countries showed that pain sensation in wound treatment is not constant, and the most acute pain is felt during removal of the dressings, followed by debridement.^[24]^ Due to the biological differences between individuals, the concentration of N_2_O required may vary in patients at different stages of dressing. Therefore, we hypothesized that N_2_O titration is more appropriate than administering fixed N_2_O concentration during burn dressing. However, studies investigating this approach in adult burn patients are limited.

This study compared the analgesic and anxiolytic effects of 50% N_2_O and N_2_O titration, to explore the efficacy and provide a rationale for the development of a model of N_2_O analgesia during burn dressing in stable patients.

## 2 Methods

### 2.1 Patients

In this single-blind prospective randomized controlled trial approved by the Fujian Medical University Union Hospital Ethics Committee (2015 ky018) and Chinese Clinical Trial Registration (ChiCTR-IPR-15006507), the subjects included hospitalized burn patients at the Burn Center of Fujian province between May 2015 and January 2016. Informed consent was obtained from all individual participants included in the study.

The inclusion criteria were: 18 to 65 years of age; NRS ≥4 during the last dressing change; burn wounds not affecting ECG monitoring; absence of burn surgery; lack of formal psychotic history, and barrier-free communication.

The exclusion criteria were: pregnancy, intestinal obstruction, air embolism, epilepsy, pneumothorax, obstructive respiratory system disease, and acute upper respiratory tract infection; ENT diseases, drug dependence, pulmonary fibrosis; exposure to opiates within 6 hours or chronically; unstable vital signs; and psychological and emotional instability.

Based on the 2 independent sample *t* test sample sizes, a significance level of 5%, and power of 90%, the dropout rate was set at 20%. According to the preliminary experimental results involving 5 cases in each group, pain was 2.42 ± 1.31 in the experimental group and 3.58 ± 1.42 in the control group during the removal of intimal dressing. A minimum of 70 patients were needed. Finally, 107 patients were recruited for the study, and 70 patients met the eligibility criteria. Following baseline evaluation in the hospital, patients were randomized by an independent researcher using an Excel-generated randomization table with 35 patients in each group. Group assignment was concealed using opaque envelopes, which were opened after the completion of the baseline assessment. In the experimental group, a patient with SpO_2_ < 90% underwent skin graft. In the control group, 2 patients were delirious and one of them was treated with additional analgesia, and excluded from the study.

**Figure 1:**
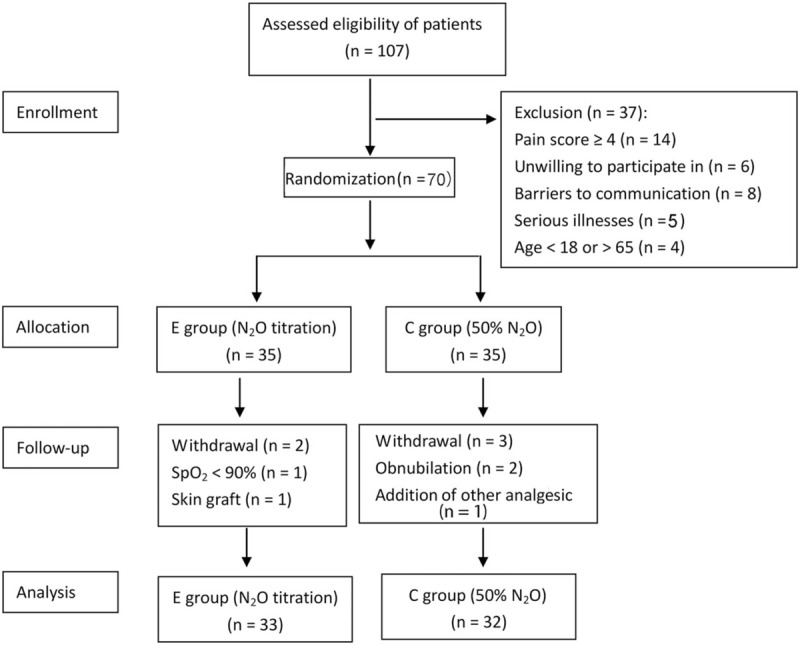
Flow chart outlining randomization of the study.

A total of 65 patients finally completed the study including 33 in the test group and 32 in the control group (Fig. 1).

### 2.2 Procedure

An effective N_2_O analgesia system (MC-AII5000C, Ambulanc, Shenzhen, China), which delivers a continuous flow of 0 to 70% was used for the intervention. Patients were covered with a mask and trained in deep breathing as well as administered 100% O_2_ for 2 minutes before dressing change. Subsequently, patients in the control group (C group) inhaled 50% N_2_O starting from 2 minutes before dressing change until the end. Patients in the experimental group (E group) inhaled 30% N_2_O initially, and the N_2_O concentration was increased from 5% to 10% every 1 to 2 minutes until patients were sedated completely. The concentration of N_2_O was adjusted by nurses based on physician recommendations to ensure optimal sedation according to the patients’ facial expression and body language. Patients’ minute ventilation remained unchanged during the procedure. Minute ventilation of patients was calculated by multiplying the tidal volume with respiratory rate. The tidal volume was determined as kg × (7 ∼ 10 mL/kg).

### 2.3 Measurements

A Numerical Rating Scale (NRS) (0–10) and Visual Analog Scale for Anxiety (VASA) were used to assess pain and anxiety, respectively, with zero indicating painless or anxiety-free condition, and 10 suggesting the worst possible pain or anxiety. The 2 scales are widely used in clinical and research studies, with high reliability and validity.^[25,26]^ We also used an ECG monitor (BeneView t6, Mindray, Shenzhen, China) to assess the vital signs. We also monitored adverse reactions of patients and inhalation of the highest concentration of N_2_O in the experimental group.

### 2.4 Outcomes

Our primary goal was to characterize and compare the pain and anxiety of patients during the T0-T6 using 50% N_2_O and N_2_O titration. The secondary outcomes included description and analysis of the highest concentration of N_2_O inhaled in the experimental group, and the vital signs and adverse reactions among the 2 groups during T0-T6.

### 2.5 Experimental protocol

Patients admitted to the hospital were randomized into groups blindly, following signed informed consent and completion of a general information questionnaire. Intervention was implemented once during days 2 to 7 of wound treatment. The whole process of dressing treatment and N_2_O analgesia was conducted by 2 doctors and 2 nurses with at least 5 years of related work experience, and who were trained in the theory and practice of N_2_O analgesia. Pain, anxiety, vital signs, side effects, and the highest concentration of N_2_O were recorded at 1 minute before N_2_O inhalation (T0); following removal of the outer dressing (T1); removal of the inner dressing (T2); debridement (T3); medication (T4); bandage (T5); and 10 minutes after dressing change (T6) by 2 other trained nurses who were nonparticipants. Doctors and nurses providing the study interventions and outcome assessors were not be blinded in this study, but they did not know the research hypothesis. According to the different dressing lengths, the measurement frequency during T1 to T5 was set to 2 to 5 minutes after each step. The final values of each step were expressed as mean (“value summation of each step" / “measuring frequency").

### 2.6 Statistical analysis

Data input was analyzed using Epidata3.0 software. SPSS19.0 was used for statistical analysis. Data analysis was done by a statistician who was blinded for the randomization allocation. Statistical significance was considered at an alpha value of 0.05 and *P* < 0.05 using a 2-tailed test. Normally distributed data were expressed as means ± SD. Otherwise, they were described using median (interquartile range [IQR]). Materials were described in medians and quartiles. Qualitative data were expressed as frequencies (percentages). Descriptive statistics, independent sample *t* test and *χ*^2^ test, Fisher exact probability method, or nonparametric tests were used to compare the general information and pain, anxiety and vital signs at T0 in both the groups. Repetitive measurement and analysis of variance (RMANOVA), and generalized estimating equations (GEEs) were used to test the interaction of intervention measures and time. RMANOVA or Wilcoxon (W) test was used to analyze data in pairs of groups. Multivariate analysis of variance (MANOVA) and Mann-Whitney *U* test were used to compare data in pairs between the two groups. *χ*^2^ was used to compare the side effects. Both primary and secondary outcomes were analyzed in each study protocol.

## 3 Results

### 3.1 Population demographics

Subjects’ age ranged from 18 to 65 years. Males accounted for 78.5% (treatment group: 84.8%, control group: 71.9%). A majority of the subjects were married (E group: 84.8%, C group: 71.9%). The education level of the 2 groups was mostly below senior middle school (E group: 75.8%; C group: 68.8%). Professionals included mainly workers and farmers (E group: 57.6%; C group: 56.3%). Patients were mostly residents in the countryside (E group: 75.8%; C group: 75.8%). Flame burns accounted for the majority of cases (E group: 69.7%; C group: 65.6%). The total burn area of the experimental group was 34.3 ± 21.5 cm^2^, compared with the control group (33.2 ± 18.7) cm^2^. Burn depth was a priority with second- and third-degree cases (E group: 84.8%; C group: 90.6%). The dressing time was within 20 minutes. No statistical significance was found in age, sex, marital status, education, occupation, income, residence, provider payments, burn area and depth, preliminary treatment time, dressing time or weight between the experimental (E), and control (C) groups (*P* > .05), as shown in Table 1.

**Table 1:**
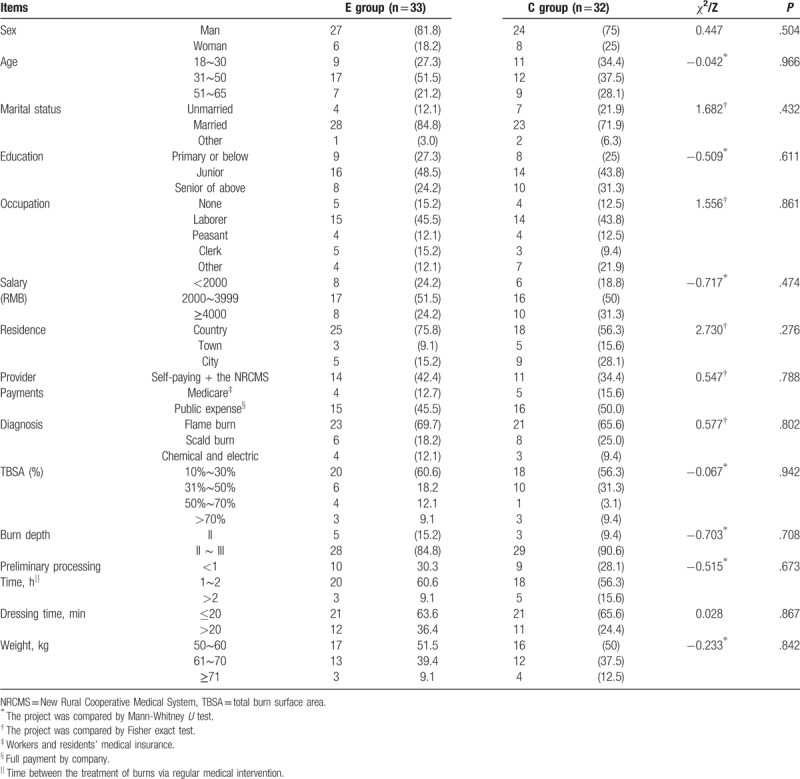
Demographics of the 2 groups.

### 3.2 Highest concentration of N_2_O in the experimental group

The N_2_O titration concentration in the experimental group ranged from 30% to 70% during T1 to T5: mainly 50% to 65% in T1 (90.9%), 60% to 70% in T2 (87.9%), T3 (87.9%), T4 (81.8%), and 40% to 60% in T5 (72.7%), as shown in Table 2.

**Table 2:**
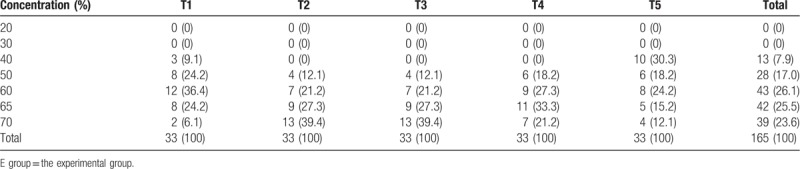
Highest concentration of N_2_O in the E group n (%).

### 3.3 Pain/anxiety

The median pain scores (NRS) were <3 in the 2 groups at T0. The anxiety scores (mean ± SD) were 4.70 ± 1.07 in the E group and 5.31 ± 1.51 in the C group at T0. The differences in pain and anxiety between the 2 groups at T0 were not statistically significant (*Z* = −4.771, *P* = .685; F = 3.596, *P* = .063).

The median pain scores of the 2 groups increased initially followed by a decline during T0 toT6. The worst pain occurred at T2 (removal of the inner dressing), which scored 3 in the E group and 4 in the C group. The pain scores of the E group were significantly lower than in the C group at T1∼T6. Pain at T2 was higher than at T0 in the E group, and pain at T1∼T4 was higher than at T0 in the C group (*Z* = −4.771∼3.767, *P* < .001).

The mean anxiety scores of the 2 groups declined significantly each time after dressing change compared with the scores before dressing (*P* < .01). The anxiety scores of E group were significantly lower than in C group at T1∼T5 (Table 3).

**Table 3:**
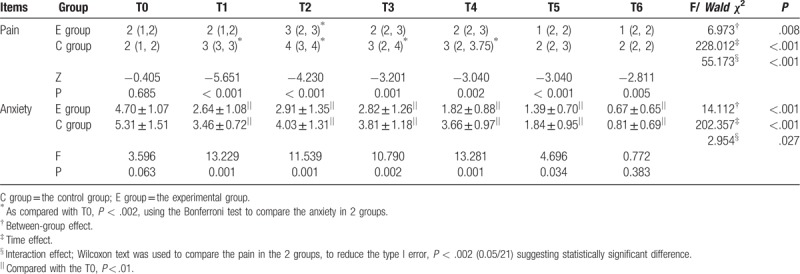
Comparison of pain/anxiety score between and in the 2 groups (x ± s)/M (Q_1_, Q_3_).

### 3.4 Vital signs

Except T2, systolic blood pressure (SBP) was not significantly different between the 2 groups at other times (*P* > .05). Furthermore, no statistically significant differences were found in the heart rate (HR), diastolic blood pressure, respiration, and blood oxygen saturation (SpO_2_) between the 2 groups at T0-T6 (Table 4).

**Table 4:**
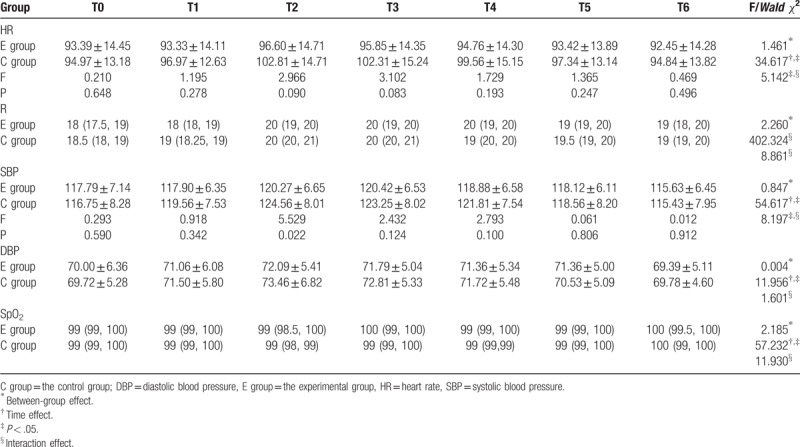
Comparison of vital signs between and in the 2 groups (x ± s)/ M (Q_1_, Q_3_).

### 3.5 Side effects

Two patients (6.1%) in the experimental group exhibited dizziness and nausea, respectively. A total of 3 patients (9.4%) showed adverse effects in the control group, including dizziness, nausea, and abnormal excitement, respectively. The differences between the 2 groups were not statistically significant (*P* > .05) (Table 5).

**Table 5:**
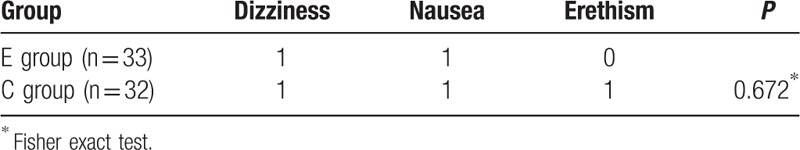
Comparison of side effects between the 2 groups.

## 4 Discussion

Dressing change is an indispensable and crucial step to stabilize the microenvironment and accelerate wound healing. However, pain during the dressing change not only directly affects the treatment, but also results in shock, affects wound rehabilitation and the quality of life.^[4]^ Furthermore, 58.2% to71.78% of the burns occur in rural China, and the primary hospital often serves as the first treatment center.^[27,28]^ Based on China's rural conditions with inadequate medical facilities and poor technological advances, along with the limitations of pain management, N_2_O undoubtedly is a safe, simple, and effective analgesia for burn patients.

Burn patients experience severe procedural pain, which is assessed by NRS without analgesia.^[1]^ However, our study found that pain ranged from mild to moderate during burn dressing change under the 2 types of N_2_O analgesia. The results are consistent with Powers et al^[29]^ who reported 61 debridement involving 46 burn patients, and found that N_2_O analgesia greatly reduced the amount of morphine consumption by patients. Our study found that the analgesic effect of N_2_O titration was better than that of 50% N_2_O because pain scores in the experimental group were lower than in the control group at T2-T6. The results may be attributed to the higher concentrations of N_2_O in the titration group at different time points especially at T2 (removal of the inner dressing) and T3 (debridement). It has been reported that 30% of N_2_O induced analgesia with 15 mg morphine administered subcutaneously.^[30]^ Therefore, every 10% of N_2_O is equivalent to the effect of 5 mg morphine administered subcutaneously. As shown in Table 2, 87.9% of patients inhaled the highest concentration of >50% up to 70% in T2 and T3 of the experimental group, which is equivalent to injection of more than 5 to 10 mg of morphine compared with the control group.

Interestingly, Liyuxiang et al^[12]^ conducted a double-blind randomized study showing that the pain scores by VAS during the burn dressing change were only 1.65 ± 1.34 versus 9.39 ± 0.74 during inhalation of 50% N_2_O in the experimental group and oxygen in the control group suggesting that the analgesic effect of 50% N_2_O was superior to the that of N_2_O titration found in our study. The pain scores in Liyuxiang's study represented the average scores during the entire dressing. Our study, by contrast, measured the pain at every step of the dressing. Therefore, we found that although 50% N_2_O was effective, the analgesic effect was not adequate during the removal of the inner dressing, debridement, and drug-smearing steps.

Pain is a complex sensation combined with sense and mood, resulting in individual variation in psychological and emotional responses. Anxiety is the most prominent and common psychological stress reaction in acute pain.^[31]^ Patients who need repeated wound management exhibit excessive anxiety due to painful dressing changes and risk of infection.^[32]^ Anxiety in turn reduces the pain threshold and increases the sensitivity to pain, leading to a vicious cycle of pain-anxiety-worse pain, which ultimately affects the condition itself.^[33]^ Therefore, accurate evaluation of anxiety in burn patients is necessary for effective analgesia.

Results showed that moderate anxiety in the 2 groups before dressing was consistent with the results reported by Tan et al.^[34]^ Physicians focus on the treatment of anxiety and pain together.^[35]^ N_2_O is a sedative and anxiolytic. Zacny et al^[36]^ showed that dental patients with high, moderate, or low anxiety showed significant enhancement in mood following N_2_O titration. A double-blind clinical trial of Manouchehrian and Bakhshaei^[37]^ showed that 50% N_2_O significantly decreased anxiety during parturition under spinal anesthesia in a cesarean section. This study also concluded that titration or 50% N_2_O effectively relieved anxiety, although the anxiolytic effect of titration was better than that of 50% N_2_O. This finding may be attributed to the larger reduction of pain and the high concentrations of laughing gas in the titration group. Starting from T1, the concentration of laughing gas, which was >50% increased in the experimental group, resulted in a stronger anti-anxiety effect.^[38]^

Severe acute pain stimulated the sympathetic nerve, promoted blood catecholamine levels and increased the angiotensin II secretion, triggering a series of organ- and tissue-specific reactions, such as accelerated HR, elevation of blood pressure, tachypnea, and increased oxygen consumption.^[39]^ Therefore, in addition to the direct evaluation, vital signs are recognized as indirect indicators of pain for the evaluation of treatment safety. N_2_O is nonirritating and does not increase airway secretions. It has almost no effect on breathing and patients exhibit a self-preservation reflex during N_2_O inhalation. Under conditions lacking oxygen, N_2_O has almost no effect on heart or blood vessels.^[40]^ Our study suggests that blood pressure, breathing, and HR varied smoothly between the 2 groups, and SpO_2_ showed almost no variation in the 2 groups, indicating safety. The SBP of the trial group in T2 was lower than in the control group. The HR of the control group at T2 and T3 exceeded 100 times/min, and was higher than in the experimental group. The differences were clinically significant. The findings suggest that N_2_O titration analgesia was better than 50% N_2_O.

The incidence of adverse reactions, which included dizziness, nausea, and excitation in the 2 groups, was lower than 10%, and consistent with previous studies. Onody et al^[41]^ showed that 50%N_2_O was most likely associated with mild side-effects including dizziness, nausea, and excitement, which ranged from 4% to 8%. The study of Zier et al^[42]^ found that adverse reactions or complications in patients who inhaled N_2_O ≤50% and >50% accounted for 1.9% and 3.5%, respectively. Most other studies showed that exposure to 50% N_2_O or N_2_O titration resulted in side effects up to 18%.^[43–45]^

### 4.1 Study limitations

The small sample size, focusing only on stable patients and single-center study, might introduce a selective bias. Multicenter and randomized controlled studies with large samples are needed.

It is a single-blind study, and the intervenors and investigators were not blinded to the study groups during dressing change and data collection, which may introduce subjective bias. Therefore, double-blind trials are needed.

This study lacked a blank control, and therefore, only indirectly evaluated the baseline analgesic effect of the 2 modes of nitrous oxide analgesia compared with other studies.

This study only conducted analgesic interventions with research subjects. The end point of observation was only 10 minutes after dressing. Therefore, the overall effects and adverse reactions subsequently or after repeated use of N_2_O were unknown. Additional follow-up studies are needed to explore the long-term role of nitrous oxide in burn patients.

## 5 Conclusions

In summary, N_2_O titration is a more appropriate intervention as analgesia during burn dressing compared with 50% N_2_O exposure. However the role of N_2_O sedation and analgesia in burn medicine in China is still at the initial stage. Further analysis of the analgesic effects in different populations (children, adults, and elderly) is needed. Appropriate protective measures (occupational exposure, treatment of exhaust air, among others) and professional operating procedures and guidelines are imperative. Relevant training in titration technology, technical expertise, and evidence-based medicine remains to be developed and improved.

## Author contributions

**Conceptualization:** Lin Li, Qiong Pan, Le Xu.

**Data curation:** Lin Li, Qiong Pan, Renqin Lin, Jiaxi Dai, Xinyan Chen, Meiyun Jiang, Zhaohong Chen.

**Formal analysis:** Lin Li, Qiong Pan, Le Xu, Renqin Lin, Jiaxi Dai, Xinyan Chen, Meiyun Jiang, Zhaohong Chen.

**Funding acquisition:** Le Xu, Xinyan Chen.

**Investigation:** Lin Li, Qiong Pan.

**Methodology:** Lin Li, Qiong Pan.

**Project administration:** Le Xu.

**Writing – original draft:** Lin Li, Qiong Pan.

**Writing – review & editing:** Le Xu, Renqin Lin, Jiaxi Dai, Xinyan Chen, Meiyun Jiang, Zhaohong Chen.

**Resources:** Zhaohong Chen, Lin Li, Le Xu.

**Software:** Lin Li, Qiong Pan.

**Supervision:** Lin Li, Le Xu

**Supervision:** Lin Li, Le Xu, Xinyan Chen

## References

[1] Esfahlan AJ Lotfi M Zamanzadeh V Burn pain and patients’ responses. Burns 2010;36:1129–33. 20471755 10.1016/j.burns.2010.02.007

[2] Yuxiang L Lingjun Z Lu T Burn patients’ experience of pain management: a qualitative study. Burns 2012;38:180–6. 22079543 10.1016/j.burns.2011.09.006

[3] Giannoni-Pastor A Eiroa-Orosa FJ Fidel Kinori SG Prevalence and predictors of posttraumatic stress symptomatology among burn survivors: a systematic review and meta-analysis. J Burn Care Res 2016;37:e79–89. 25970798 10.1097/BCR.0000000000000226

[4] Bell L Pain scales and pain management. Am J Crit Care 2012;21:260. 22751368 10.4037/ajcc2012599

[5] Ferguson SL Voll KV Burn pain and anxiety: the use of music relaxation during rehabilitation. J Burn Care Rehabil 2004;25:8–14. 14726734 10.1097/01.BCR.0000105056.74606.9E

[6] Richardson P Mustard L The management of pain in the burns unit. Burns 2009;35:921–36. 19505764 10.1016/j.burns.2009.03.003

[7] Byers JF Bridges S Kijek J Burn patients’pain and anxiety experiences. J Burn Care Rehabil 2001;22:144–9. 11302603 10.1097/00004630-200103000-00011

[8] James DL Jowza M Principles of burn pain management. Clin Plast Surg 2017;44:737–47. 28888299 10.1016/j.cps.2017.05.005

[9] Gregorian RS Kavanagh S Importance of side effects in opioid treatment: a trade off analysis with patients and physicians. J Pain 2010;11:1095–108. 20452835 10.1016/j.jpain.2010.02.007

[10] Marret E Kurdi O Zufferey P Effects of nonsteroidal antiinflammatory drugs on patientcontrolled analgesia morphine side effects: meta-analysis of randomized controlled trials. Anesthesiology 2005;102:1249–60. 15915040 10.1097/00000542-200506000-00027

[11] Giessler GA Mayer T Trupkovic T Das Verbrennungstrauma. Anaesthesist 2009;58:474–84. 19384454 10.1007/s00101-009-1535-y

[12] Yuxiang L Wenjun H Hongtai T Nitrous oxide-oxygen mixture during burn wound dressing: a double-blind randomized controlled study. CNS Neurosci Ther 2013;19:278–9. 23406362 10.1111/cns.12061 PMC6493482

[13] Clark M Brunick A Handbook of nitrous oxide and oxygen Sedation. 2nd ed. 2003;UK: Mosby Elsevier, 1–256.

[14] Dammer U Weiss C Raabe E Introduction of inhaled nitrous oxide and oxygen for pain management during labour—evaluation of patients’ and midwives’ satisfaction. Geburtshilfe Frauenheilkd 2014;74:656–60. 25100880 10.1055/s-0034-1368606 PMC4119100

[15] Singh RH Espey E Carr S Nitrous oxide for pain management of first trimester surgical abortion -- a randomized controlled pilot study. Contraception 2015;91:164–6. 25459096 10.1016/j.contraception.2014.09.013 PMC5317271

[16] Fassoulaki A Staikou C Pretreatment with nitrous oxide enhances induction of anesthesia with sevoflurane: a randomized controlled trial. J Anaesthesiol Clin Pharmacol 2015;31:511–6. 26702210 10.4103/0970-9185.169079 PMC4676242

[17] Kurien T Price KR Pearson RG Manipulation and reduction of paediatric fractures of the distal radius and forearm using intranasal diamorphine and 50% oxygen and nitrous oxide in the emergency department: a 2.5-year study. Bone Joint J 2016;98-B:131–6. 26733526 10.1302/0301-620X.98B1.36118

[18] Takkar D Rao A Shenoy R Evaluation of nitrous oxide inhalation sedation during inferior alveolar block administration in children aged 7-10 years: a randomized control trial. J Indian Soc Pedod Prev Dent 2015;33:239–44. 26156280 10.4103/0970-4388.160399

[19] Mattos Junior FM Mattos RV Teixeira MJ Chronic pain relief after the exposure of nitrous oxide during dental treatment: longitudinal retrospective study. Arq Neuropsiquiatr 2015;73:578–81. 26200051 10.1590/0004-282X20150061

[20] Luhmann JD Kennedy RM Porter FL A randomized clinical trial of continuous-flow nitrous oxide and midazolam for sedation of young children during laceration repair. Ann Emerg Med 2001;37:20–7. 11145766 10.1067/mem.2001.112003

[21] Baskett PJ Analgesia for the dressing of burns in children: a method using neuroleptanalgesia and Entonox. Postgrad Med J 1972;48:138–42. 5024151 PMC2495215

[22] Kanagasundaram SA Lane LJ Cavalletto BP Efficacy and safety of nitrous oxide in alleviating pain and anxiety during painful procedures. Arch Dis Child 2001;84:492–5. 11369566 10.1136/adc.84.6.492 PMC1718795

[23] do Vale AH Videira RL Gomez DS Effect of nitrous oxide on fentanyl consumption in burned patients undergoing dressing change. Braz J Anesthesiol 2016;66:7–11. 26768923 10.1016/j.bjane.2014.07.016

[24] Hollinworth H How to alleviate pain at wound dressing changes. Nurs Times 2002;98:51–2. 12451750

[25] Yang HT Hur G Kwak IS Improvement of burn pain management through routine pain monitoring and pain management protocol. Burns 2013;39:619–24. 23182650 10.1016/j.burns.2012.10.025

[26] Park E Oh H Kim T The effects of relaxation breathing on procedural pain and anxiety during burn care. Burns 2013;39:1101–6. 23375536 10.1016/j.burns.2013.01.006

[27] Sun CF Lv XX Li YJ Epidemiological studies of electrical injuries in Shaanxi province of China: a retrospective report of 383 cases. Burns 2012;38:568–72. 22103989 10.1016/j.burns.2011.10.012

[28] Liu H Liu F Xiao S Epidemiological characteristics of burn workers in Hunan Province. Zhong Nan Da Xue Xue Bao Yi Xue Ban 2014;39:84–90. 24473377 10.11817/j.issn.1672-7347.2014.01.015

[29] Powers PS Cruse CW Daniels S Safety and efficacy of debridement under anesthesia in patients with burns. J Burn Care Rehabil 1993;14:176–80. 8501106 10.1097/00004630-199303000-00009

[30] Masood J Shah N Lane T Nitrous oxide (Entonox) inhalation and tolerance of transrectal ultrasound guided prostate biopsy: a double-blind randomized controlled study. J Urol 2002;168:116–20. discussion 120. 12050503

[31] Trame CD Greene E Moddeman G Correlation of pain scores, analgesic use, and beck anxiety inventory scores during hospitalization in lower extremity amputees. Open Orthop J 2008;2:145–50. 19478937 10.2174/1874325000802010145 PMC2687119

[32] Hsu KC Chen LF Hsiep PH Effect of music intervention on burn patients’ pain and anxiety during dressing changes. Burns 2016;42:1789–96. 27263418 10.1016/j.burns.2016.05.006

[33] Lehofer M Liebmann PM Moser M Nervousness and pain sensitivity: I. A positive correlation. Psychiatry Res 1998;79:51–3. 9676826 10.1016/s0165-1781(98)00023-7

[34] Tan X Yowler CJ Super DM The efficacy of music therapy protocols for decreasing pain, anxiety, and muscle tension levels during burn dressing changes: a prospective randomized crossover trial. J Burn Care Res 2010;31:590–7. 20498613 10.1097/BCR.0b013e3181e4d71b

[35] Holtman JR Jr Jellish WS Opioid-induced hyperalgesia and burn pain. J Burn Care Res 2012;33:692–701. 23143613 10.1097/BCR.0b013e31825adcb0

[36] Zacny JP Hurst RJ Graham L Preoperative dental anxiety and mood changes during nitrous oxide inhalation. J Am Dent Assoc 2002;133:82–8. 11811748 10.14219/jada.archive.2002.0026

[37] Manouchehrian N Bakhshaei MH Nitrous oxide effect on relieving anxiety and pain in parturients under spinal anesthesia for caesarean section. Anesth Pain Med 2014;4:e16662. 24977119 10.5812/aapm.16662 PMC4071269

[38] Cote CJ Wilson S American Academy of Pediatrics; American Academy of Pediatric Dentistry. Guidelines for monitoring and management of pediatric patients during and after sedation for diagnostic and therapeutic procedures: an update. Pediatrics 2006;118:2587–602. 17142550 10.1542/peds.2006-2780

[39] Seol TK Lim JK Yoo EK Propofol-ketamine or propofol-remifentanil for deep sedation and analgesia in pediatric patients undergoing burn dressing changes: a randomized clinical trial. Paediatr Anaesth 2015;25:560–6. 25557125 10.1111/pan.12592

[40] Okushima K Kohjitani A Asano Y Inhalational conscious sedation with nitrous oxide enhances the cardiac parasympathetic component of heart rate variability. Oral Surg Oral Med Oral Pathol Oral Radiol Endod 2008;106:e1–5. 10.1016/j.tripleo.2008.08.020 19000603

[41] Onody P Gil P Hennequin M Safety of inhalation of a 50% nitrous oxide/oxygen premix: a prospective survey of 35 828 administrations. Drug Saf 2006;29:633–40. 16808555 10.2165/00002018-200629070-00008

[42] Zier JL Liu M Safety of high-concentration nitrous oxide by nasal mask for pediatric procedural sedation: experience with 7802 cases. Pediatr Emerg Care 2011;27:1107–12. 22134227 10.1097/PEC.0b013e31823aff6d

[43] Meier TO Jacomella V Clemens RK Nitrous oxide/oxygen inhalation provides effective analgesia during the administration of tumescent local anaesthesia for endovenous laser ablation. Vasa 2015;44:473–8. 26515225 10.1024/0301-1526/a000471

[44] Paracka L Kollewe K Dengler R Botulinum toxin therapy for hyperhidrosis: reduction of injection site pain by nitrous oxide/oxygen mixtures. J Neural Transm (Vienna) 2015;122:1279–82. 25645865 10.1007/s00702-015-1372-x

[45] Del Valle Rubido C Solano Calvo JA Rodriguez Miguel A Inhalation analgesia with nitrous oxide versus other analgesic techniques in hysteroscopic polypectomy: a pilot study. J Minim Invasive Gynecol 2015;22:595–600. 25596171 10.1016/j.jmig.2015.01.005

